# Development of Porous-Polyacrylonitrile-Based Fibers Using Nanocellulose Additives as Precursor for Carbon Fiber Manufacturing

**DOI:** 10.3390/polym15030565

**Published:** 2023-01-21

**Authors:** Iris Kruppke, Fawzy Sherif, Mirko Richter, Chokri Cherif

**Affiliations:** 1 Institute of Textile Machinery and High Performance Material Technology (ITM), Technical University Dresden (TUD), 01069 Dresden, Germany; 2 Research Center Carbon Fibers Saxony (RCCF), Technische Universität Dresden, 01237 Dresden, Germany; 3 Department of Clothing and Textiles, Faculty of Home Economis, Menoufia University, Shibin El Kom 6131567, Menofia Governorate, Egypt

**Keywords:** nanocellulose, polyacrilonitrile, wet spinning, precursors, porous fibers, process development

## Abstract

Cellulose is a renewable and environmentally friendly raw material that has an important economic and technical impact in several applications. Recently, nanocellulose (NC) presented a promising road to support the manufacturing of functional carbon fibers (CFs), which are considered superior materials for several applications because of their outstanding properties. However, the smooth and limited effective surface areas make CFs virtually useless in some applications, such as energy storage. Therefore, strategies to increase the porosity of CFs are highly desirable to realize their potential. Within this article, we present an approach that focuses on the designing of porous CF precursors using polyacrilonitrile (PAN) and NC additives using a wet spinning method. To enhance the porosity, two jet stretching (50% and 100%) and four NC additive amounts (0 wt.%, 0.1 wt.%, 0.4 wt.% and 0.8 wt.%) have been applied and investigated. In comparison with the reference PAN fibers (without NC additives and stretching), the results showed an increase in specific surface area from 10.45 m^2^/g to 138.53 m^2^/g and in total pore volume from 0.03 cm^3^/g to 0.49 cm^3^/g. On the other hand, mechanical properties have been affected negatively by NC additives and the stretching process. Stabilization and carbonization processes could be applied in a future study to support the production of multifunctional porous CF.

## 1. Introduction

Carbon fibers (CFs) contain more than 90 wt.% of carbon and exhibit many outstanding properties such as high modulus (200–900 GPa), high compressive strength (up to 3 GPa), high tensile strength (2–7 GPa) [1,2], flexibility and tunable electrochemical performance. Therefore, they can be used in multiple fields of applications [3], such as aerospace, automobile, the chemical industry, transportation, construction, energy storage, sewage treatment and others [4]. The first step of a CF production process is to select the precursor material that determines the properties and applications of CF produced from it [5]. Numerous petrol-based precursors can be used, such as polyacrylonitrile (PAN), pitch and polyolefins [6]. CFs are mainly manufactured from PAN, which is dissolved in a polar, often organic, solvent and wet-spun into precursor filaments. PAN-based CFs have excellent mechanical properties, but there is a major drawback: they are very expensive and, therefore, limited to applications where cost is not a major factor [7,8].

Typically, nanocellulose (NC) can be categorized into two major classes. The first is nanostructured materials (cellulose microcrystals and cellulose microfibrils), and the second is nanofibers (cellulose nanofibers (CNFs), cellulose nanocrystals (CNCs) and bacterial cellulose) [9,10,11].

Jiang et al. reported the influence of three different NC types on the mechanical properties and thermal transformations of solution-spun PAN/NC nanocomposite fibers into CF. Therefore, the derivatization method, the source and the aspect ratio of NC in the PAN solution were varied. In this study, the NC aspect ratio and crystallinity were found to be inversely related. The increase in the CF mechanical properties was also related to a decrease in the crystallinity of the NC additives [12]. In another study, Jiang et al. investigated the rheological and electrospinning properties of PAN/CNF composite fibers. The solutions depended on different CNF concentration levels, and the fibers were subsequently stabilized and carbonized. They proved that CNF loadings of 0.5–2 wt.% resulted in spinnable solutions with well-ordered carbon structures, which exhibit a reduced Raman D/G ratio and an increased band intensity by XRD [13].

CNCs have attracted considerable attention as a renewable reinforcing material for polymer nanocomposites due to their high specific tensile strength and modulus, uniform orientation and ecofriendly and biodegradable nature [14,15]. The most important methodology to produce CNCs is commonly based on acid hydrolysis, although currently, several other procedures have been established [16]. In terms of PAN-based composite fibers containing CNCs (with a maximum of 2% CNC loading), Park et al. have demonstrated improvements in the tensile strength and the modulus in both the wet-spun precursor fiber and that after carbonization at 1000 °C [17]. In another study, Chang et al. demonstrated mechanical property enhancements achieved in a PAN/CNC CF, comparable to those of a pure PAN fiber, highlighting the potential for more bio-based CF [18].

Luo et al. studied the stabilization kinetics of PAN-co-methacrylic acid (PAN-co-MAA) and CNC composite films with up to 40 wt.% CNC loading using differential scanning calorimetry (DSC). It was determined that, by adding CNCs, the activation energy of the PAN cyclization reaction was reduced by 16%, irrespective of the CNC loading in the range of 5 to 40 wt.%. In contrast, the activation energy of oxidation was minimally affected by CNC additions. They summarized that the oxidation reaction of PAN-co-MAA was influenced by the surface area to volume ratio of the tested sample, while the cyclization reaction was unaffected [19].

In order to prepare PAN fibers that contain fine cellulose particles, it is important to create a uniformly dispersed spinning dope. The minimization of the cellulose particle size was performed through a heat treatment at various temperatures in order to reduce the cohesive forces from the hydrogen bonds between the cellulose molecules. Yang et al. used heat treating of cellulose microparticles at 400 °C as a method for the dispersion in the PAN spinning dope and studied their effects of cellulose particles on the PAN dope. Based on the results, they found that, from examining the dispersion characteristics of the spun fibers with carbonized cellulose, the condition of carbonization at 400 °C revealed a relatively good dispersion of the cellulose particles in the dope [20]. By using gel spinning, Chang et al. produced PAN/CNC fibers, which contain between 0 wt.% and 10 wt.% CNCs with DMF as a solvent. Regarding the rheological properties of the PAN solution, this study showed that the solution behavior changes from Newtonian fluid to a shear-thinning behavior with the presence of CNCs. It also showed that tensile modulus and strength increased from 14.5 to 19.6 GPa and from 624 to 709 MPa, respectively, as CNC loading increased from 0 wt.% to 10 wt.% [21]. The benefits of the 3D hierarchical nanostructure of NC and its physicochemical characteristics at nanoscale open new prospects in several applications [22,23,24,25]. Remarkably, cellulosic materials with an ordered porous structure are highly favorable for the development of a new range of applications.

Next to the presented cellulose-based additives for precursors, other methods can be used to ensure a porous structure in precursor fibers and, consequently, in carbon nanofibers and CFs. Different studies showed that the addition of additives such as chloroform (CHCl_3_), tertrahydrofuran (THF), graphene oxide (GO) or lignin to the spinning solution [26,27,28] or chemical activation of preoxidized commercial PAN with potassium hydroxid (KOH) [5] resulted in CFs with surface areas reaching from 31.8 m^2^/g to 2231 m^2^/g and pore volumes between 0.07 cm^3^/g and 1.16 cm^3^/g. In addition, Li et al. fabricated an interpenetrating 3D hierarchical porous CF doped with numerous nitrogen and oxygen atoms depending on PAN wet spinning in a coagulation bath with dimethyl sulfoxide/water. The obtained structure surface was 2176.6 m^2^/g with a pore volume of 1.272 cm^3^/g. This process has been considered promising for the production of porous precursors for CFs [29].

NC-based materials are now recognized as unique materials that can be used to produce exceptional films, composites and gels that show interesting features as an alternative to petroleum-based materials with environmentally friendly and renewable characteristics. Most of the previous studies focus on using PAN with other chemicals to produce precursors with high mechanical properties for several high-performance applications. However, few studies highlight the importance of porous precursors for CF manufacturing, especially in the case of utilizing natural resources involved in that process, not only for applications that need a high amount of mechanical properties but mainly for applications that need porosity properties, such as energy storage. NC is a versatile nanomaterial with tailorable surface functionalities, dimensions and morphologies and offers a potentially low-cost, bio-based alternative additive for PAN-based CFs. Therefore, the main goal of this work is to develop porous PAN-based precursors using NC additives with the intention of manufacturing porous CFs. The modification of the PAN precursor fiber using nanoadditives offers a promising strategy for the development of multifunctional porous CFs. We investigated the effect of a combination of NC addition and supercritical (disproportionately) stretching processes during the wet spinning on the morphology and mechanical properties to produce porous PAN/NC fibers. 

The experimental part of this study investigates the wet spinning technique, which has been considered as a preferred method to obtain high-strength PAN-based fibers. It is worth noting here that this study highlights the production of porous PAN/NC precursors in a wet spinning process, which is the most important process in the manufacturing of CFs. Carbonization and stabilization processes will be applied and investigated during a future study.

## 2. Materials and Methods

### 2.1. Materials

In this work, PAN homopolymer powder (DOLAN GmbH, Kelheim, Germany) with at least 99.5 wt.% of acrylonitrile was used to produce porous precursor fibers. Dimethylformamide (DMF) produced by Brenntag GmbH (Mülheim, Germany) was used as solvent. The NC additive, depending on CNCs, was obtained by Celluforce Company, Montreal, QC, Canada (commercial type: Celluforce NCV100-NASD90).

### 2.2. Spinning Solutions

Herein, we report the production of porous precursors for CFs that depends on four different ratios of NC additives (0 wt.%, 0.1 wt.%, 0.4 wt.% and 0.8 wt.%) to PAN in DMF as a solvent. For the production of the reference fiber, a solution of 18 wt.% PAN and 82 wt.% DMF was stirred for 3 h at 70 °C and was used to spin the reference sample (without NC additives). In the case of the PAN/NC/DMF solutions, NC was ultrasonic-treated at an output power of 100 W and a frequency of 37 kHz for 30 min in DMF. Then, PAN was added and stirred for 3 h at 70 °C in order to spin the PAN/NC samples. Three PAN/NC/DMF solutions were prepared depending on the ratio of NC as mentioned.

### 2.3. Wet Spinning and Stretching

The fiber spinning was performed at a wet spinning pilot plant (Fourné Polymertechnik GmbH, Alfter-Impekoven, Germany) consisting of one coagulation bath, three washing baths and one drying and one sizing unit as well as a winder. The spinning solutions were heated until 70 °C, extruded through a filter (50 μm) and spun with a spinneret with 1008 holes of 70 μm diameter. The wet spinning process of PAN/NC/DMF solutions depended on a PAN content of 18 wt.% and a coagulation bath of DMF/water with a concentration of 15 wt.% water at 30 °C.

Several parameters were adapted during the process such as tank pressure, washing baths temperatures, speed of godets and other spinning parameters to improve and stabilize the spinning process. The produced fibers were dried at a temperature between 45 and 50 °C. During the fiber spinning, two different stretching ratios were used for each fiber composition in order to investigate the effect of this process on the properties of the produced porous fibers. The two supercritical stretching ratios were related to the used stretching for the reference. For the first series of PAN/NC fibers, the stretching was 1.5 times the stretching ratio compared with the reference (an additional 50%), and for the second series, the stretching ratio was double the reference (an additional 100%). Table 1 describes the ratios of NC additives and stretching for each sample.

### 2.4. Characterization

Rheological characterization of all solutions was conducted using a HAAKE MARS Rheometer (Thermo Fischer Scientific Company, Waltham, MA, USA) in a parallel plate geometry, with a 35 mm plate on top, a Peltier plate on the bottom and a 1 mm gap size in between. The rheological experiments were conducted in the frequency range from 1.0 to 100 Hz at 40 °C. To measure the diameter of the fibers, the mounting of fiber specimens was prepared with a thermosetting resin. Afterwards, the mounted specimens were ground using a finer paper. The fiber cross-sections were imaged and measured by an Axio Imager.M1m Microscope (ZEISS GmbH, Jena, Germany).

The surface morphology of the precursor fiber shells and the cross-sectional areas were characterized by using scanning electron microscopy (SEM). For this, the fibers were cooled with liquid nitrogen and cut manually. Subsequently, the fiber segments were fixed on carbon pads and sputter-coated with gold. The electron images were taken using a Philips ESEM XL 30 SEM (Amsterdam, The Netherlands). Representative images at magnifications of 10,000× and 20,000× were selected.

For the quantification of the pore volumes and surface areas, nitrogen physisorption measurements were performed at 77 K on a NOVA 4000e (Quantachrome Instruments, Boynton Beach, FL, USA) after degassing the samples under vacuum for 12 h at room temperature. The specific surface area was calculated with the multipoint Brunauer–Emmett–Teller (BET) method in the range of relative pressure of p/p0 = 0.05–0.3, and the total pore volume was determined at p/p0 = 0.97. The pore size distribution (PSD) measurements were based on nitrogen adsorption isotherms using the QSDFT model (slit/cylindrical pores) implemented in the ASiQwin software (Quantachrome Instruments, United States). Finally, fineness strength, elongation and Young’s modulus properties of the fibers were measured using a single fiber tester (FAVIMAT, Textechno Herbert Stein GmbH & Co. KG, Mönchengladbach, Germany) according to DIN DIN EN ISO 5079. The same properties but in the case of yarns have been tested using zwickiLine device (ZwickRoell GmbH & Co. KG, Ulm, Germany) according to DIN EN ISO 2062.

## 3. Results and Discussion

### 3.1. Rheological Investigations of the Used Spinning Solutions

**Figure 1:**
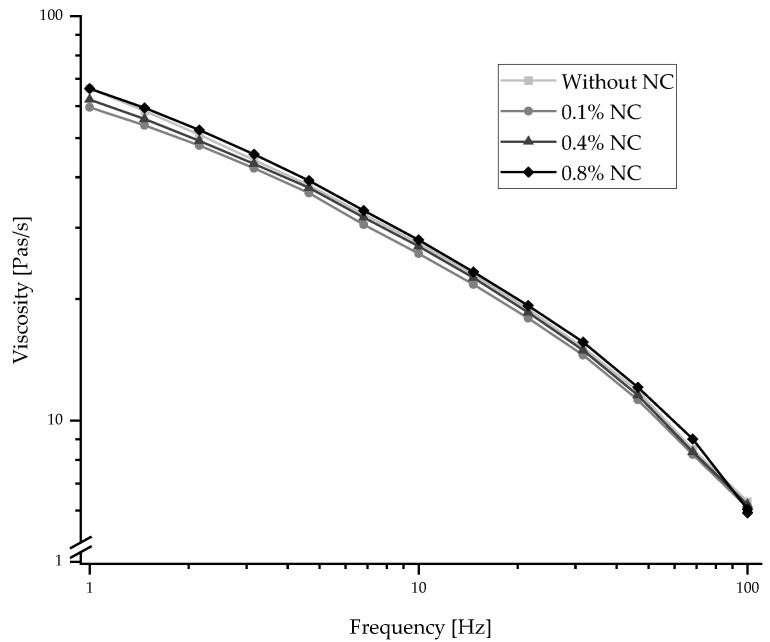
Viscosity of the PAN/NC spinning solutions.

Figure 1 describes the frequency-dependent viscosity of the PAN solutions with and without NC addition. The different solutions show quite similar properties. The highest values of viscosities can be found at low levels of frequency, and with increasing frequency, the viscosity decreases. In general, the NC additives also change the viscosity. It decreases to the lowest value after adding 0.1 wt.% NC to the spinning solution. By adding 0.4 wt.% NC, the viscosity decreases less. The highest value of viscosity shows the solution with 0.8 wt.% NC, which is similar to the PAN solution without any NC additives. That means that NC additives until 0.8 wt.% could reduce the viscosity of PAN spinning solution because the NC disturbs the strong intermolecular dipole–dipole interactions between the nitrilie groups of the PAN chains [30,31]. On the other hand, the intermolecular interactions (hydrogene bonds) between the NC molecules increase with increasing NC content [31]. This leads to an increasing viscosity, again.

For the following spinning of the PAN/NC fibers, the rheological measurements showed that the solutions with the chosen NC content range can be spun with similar parameters as the reference PAN spinning solution.

### 3.2. Influence of NC Additives and Stretching Process on Morphology of the Fibers

For the production of commercial CF precursors, wet spinning is still the most commonly used technique today [32]. Here, the precipitation of the fibers in the coagulation bath is the decisive process step that affects the fiber shape, imperfections and voids in particular. The crucial process parameters to adjust the resulting precursor fiber properties are the temperature and the concentration of the coagulation bath [6]. When the PAN/DMF jet leaves the spinneret to enter into the coagulation bath (DMF/water), the DMF (solvent) diffuses out of the fiber, while the water (nonsolvent) diffuses into it due to the concentration difference between the fiber and the coagulation bath. Disturbing the coagulation process by the presence of NC as a pore-building agent and, additionally, purposefully uncommon stretching parameters was the concept of this study to build the porous fibers. This should lead to a highly disturbed, porous morphology. Initially, the diameters of the developed porous fibers have been affected by NC additives ratios and the stretching process (Table 1). It increases between 15.2 and 29.8% in comparison with the reference PAN. The lowest diameter of 14.73 µm is for the reference PAN fibers. The highest diameter of 20.96 µm is for the fibers with 0.8 wt.% NC addition and 100% stretching. The increase in diameter is caused by the addition of the NC. While water is important for the process as a dissolvent for PAN, it additionally affects the NC. The water molecules form hydrogen bonds to the hydroxyl groups of the NC, which leads to a swelling of the NC [33,34]. This swelling increases the fiber diameter. Therefore, in general, the increasing NC content enlarges the diameter of the fibers.

The expected effect of stretching during the fiber spinning process is a reduction in the diameter [35]. This reduction can be seen by comparing the diameter of the PAN fibers without NC at 50% and 100% stretching. In contrast, for the fibers with NC, the diameter enlarges at higher stretching ratios. Stretching not only creates a more uniform orientation of the PAN chains but also causes chain ripping, as the chosen stretching ratios are supposed to be supercritical [35]. The breaking points are pore-forming agents in the further stretching process. Therefore, it can be assumed that the porosity increases at higher stretching ratios. This porosity allows a better diffusion out of the fibers and into the fibers of DMF and water, respectively. Therefore, higher swelling ratios of the NC can be expected, which lead to larger diameters at higher stretching ratios in the presence of NC.

The increase in the diameter of the PAN fibers without NC due to stretching compared with the reference fibers, on the other hand, can be explained by the previously described adaption of the spinning parameters.

**Figure 2:**
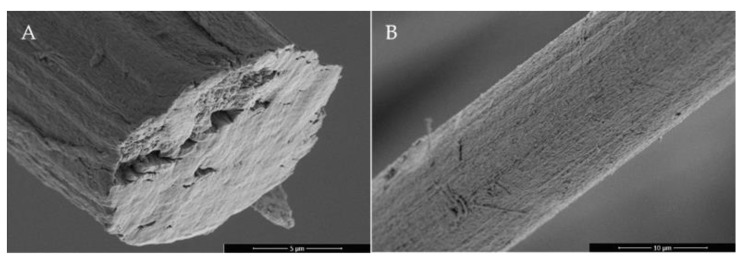
SEM images of the cross-section (**A**) and the surface (**B**) of the control fibers without stretching and NC addition.

**Figure 3:**
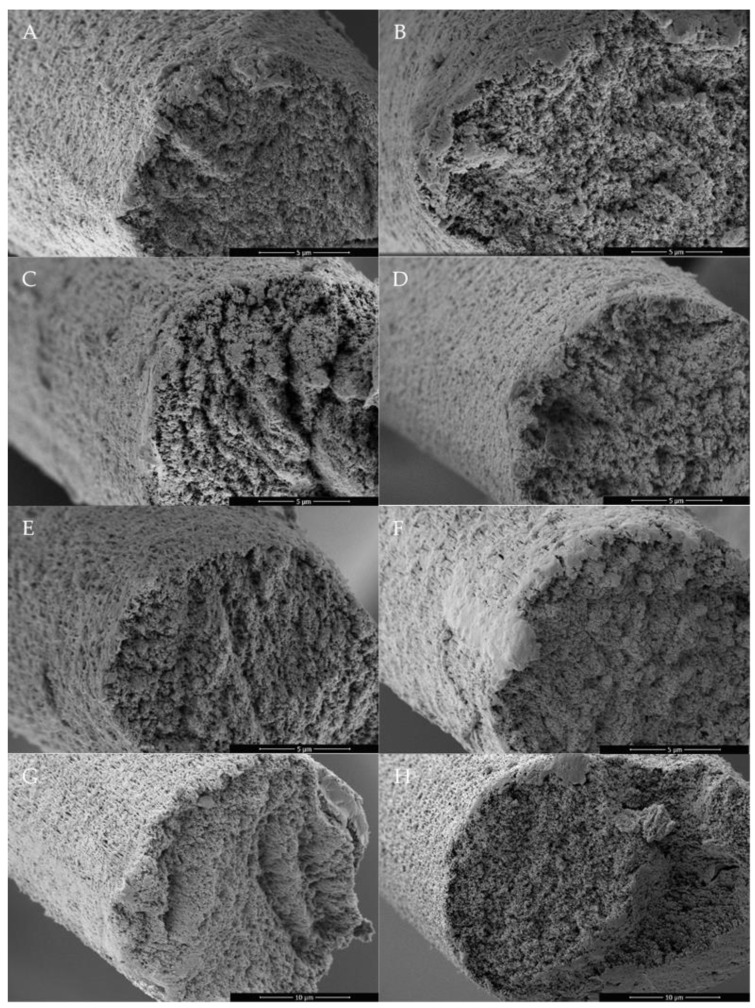
SEM images of the cross-section of the developed porous PAN/NC fibers after 50% stretching (left): (**A**) 0 wt.% NC, (**C**) 0.1 wt.% NC 1, (**E**) 0.4 wt.% NC 4 and (**G**) 0.8 wt.% NC; 100% stretching (right): (**B**) 0 wt.% NC, (**D**) 0.1 wt.% NC 1, (**F**) 0.4 wt.% NC 4 and (**H**) 0.8 wt.% NC.

**Figure 4:**
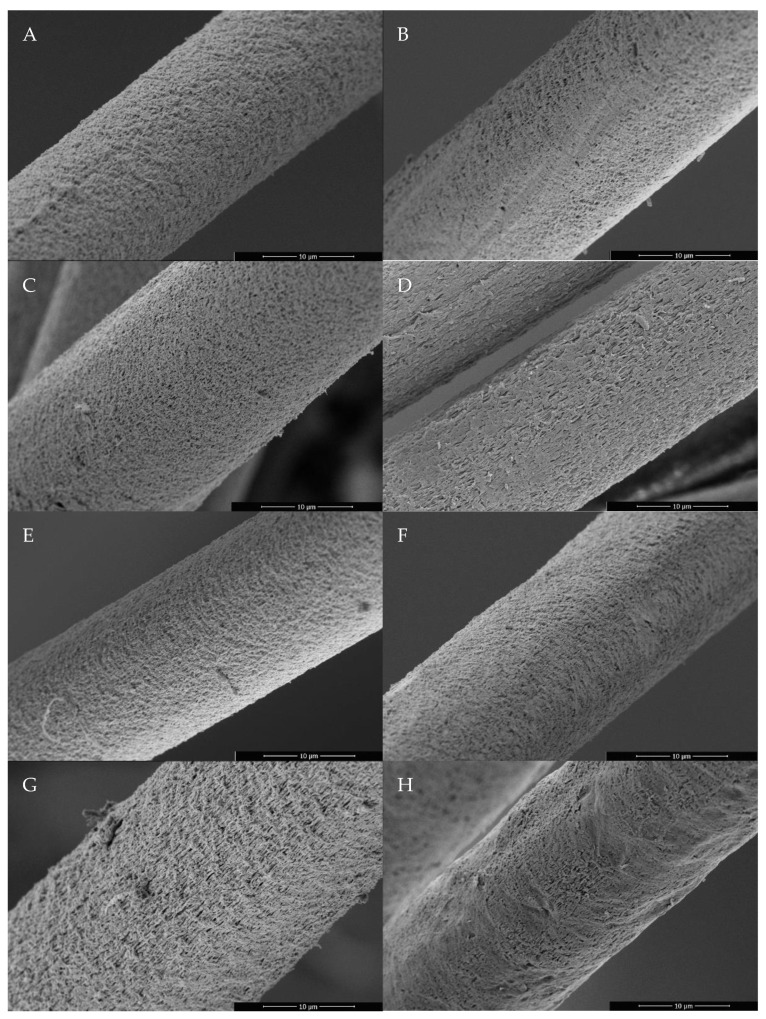
SEM images of the surface of the developed porous PAN/NC fibers after 50% stretching (left): (**A**) 0 wt.% NC, (**C**) 0.1 wt.% NC 1, (**E**) 0.4 wt.% NC 4 and (**G**) 0.8 wt.% NC; 100% stretching (right): (**B**) 0 wt.% NC, (**D**) 0.1 wt.% NC 1, (**F**) 0.4 wt.% NC 4 and (**H**) 0.8 wt.% NC.

As seen in Figure 2 in comparison to Figure 3 and Figure 4, there is a clear difference in the cross-section and surface between the reference PAN fiber and the other developed porous PAN/NC fibers. The cross-section of the reference PAN fiber (Figure 2A) is more closed in comparison with the developed porous PAN/NC fibers (Figure 3). It can be assumed that the uneven cross-sections of these fibers are caused by the pores created by stretching and the NC addition. 

The surface of the reference PAN fiber (Figure 2B) is also smoother than the surface of the developed porous PAN/NC fibers (Figure 4). However, the surfaces of the fibers, which were spun with a stretching ratio of 100% (Figure 4B,D,F,H), show local smoother areas. This leads to the conclusion that the PAN chains in these regions are high-oriented and less disturbed by the pores.

### 3.3. Pore Volume and Surface Area Measurements

**Figure 5:**
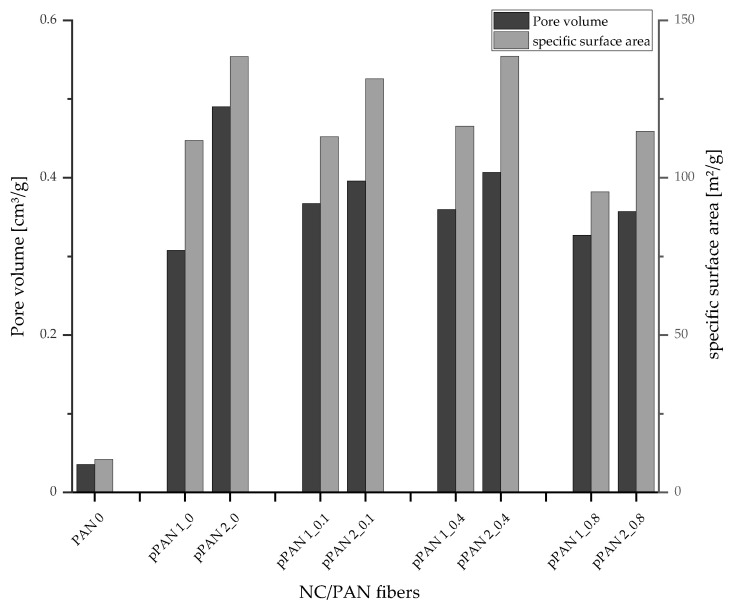
Total pore volume and specific surface area of the developed porous PAN/NC fibers.

Depending on BET and DFT methodology, the N_2_ adsorption measurements for the produced porous PAN/NC fibers were evaluated. Figure 5 shows the great difference in the values of surface area and total pore volume between the reference sample (without stretching and NC additives) and the developed porous PAN/NC fibers. The surface area of the developed porous PAN/NC increases by a factor between 9.14 and 13.26 in comparison with the reference PAN surface area, while the total pore volume increases by a factor between 10.0 and 16.3. The reference PAN surface area and total pore volume are 10.45 m^2^/g and 0.03 cm^3^/g, respectively. In comparison, the developed porous PAN/NC fibers have surface areas between 95.49 m^2^/g and 138.53 m^2^/g and total pore volumes between 0.30 cm^3^/g and 0.49 cm^3^/g.

Comparing the different stretching ratios, there is a difference among the developed porous fibers. The surface area and total pore volume increase more in the case of 100% stretching than in the case of 50% stretching. The higher forces at 100% stretching disturb the fiber coagulation more effectively and force more gaps within the built filament, which leads to a more porous structure.

Regarding the NC content, the highest values of the surface area were measured at 0.4 wt.% NC. At higher and lower NC ratios, the surface areas decrease except in the case of 0% NC and 100% stretching, where the surface area rises again (compared to 0.1 wt.% NC and 100% stretching). The same tendencies can be seen for the total pore volume. The difference is that, in the case of 100% stretching, the pore volume of the fibers with 0% NC is even higher than the one of the fibers with 0.4 wt.% NC.

As explained before, two opposing effects can be identified, concerning their influence on the surface area and the total pore volume: on the one hand, the NC and stretching disturb the chain formation of the PAN and create pores in the structure. But on the other hand, the swelling of the NC closes pores. At an NC content of 0.4 wt.% the effects reach an equilibrium in terms of pore volume and surface area in the case of the chosen NC ratios. At higher NC ratios, the swelling of the NC closes more pore volume than the NC can create in the PAN structure. At lower NC ratios, the NC creates less pore volume. However, in the case of 0% NC and 100% stretching, the effect of stretching is higher than the combined effect of stretching and NC addition reduced by NC swelling in the case of 0.4 wt.% NC and 100% stretching.

### 3.4. Influences of NC Additives and Stretching Process on Mechanical Properties

As mentioned in the experimental part, this study did not apply carbonization or stabilization processes for the developed porous fibers. The main target is the development of porous PAN/NC fibers by using a wet spinning process. With a successful outcome, further research in that field is likely to happen. Several mechanical properties have been tested for the developed porous fibers, such as fineness strength, elongation and Young’s modulus.

**Figure 6:**
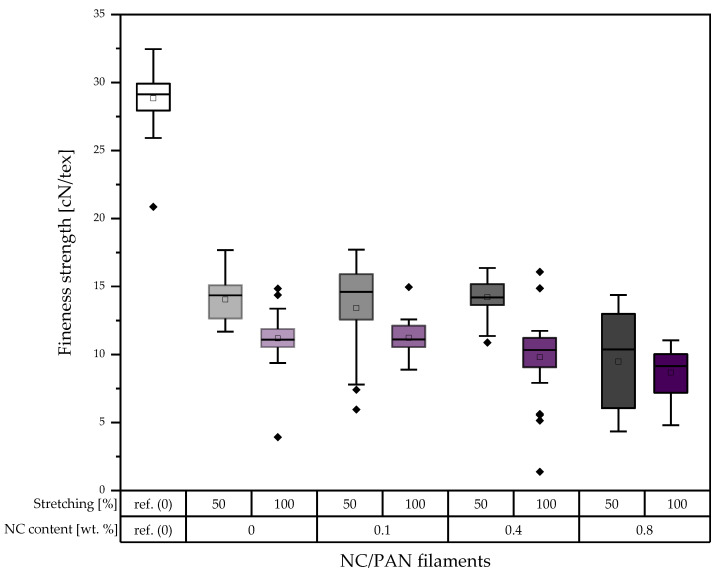
Fineness strength of the developed porous PAN/NC filaments (in the boxes: sorted values between 25% and 75%; between the whiskers: values within 1.5 · IQR; central line: median; square: mean; and rhombus: outlier).

Comparing the fineness strength of the developed porous PAN/NC filaments to the reference PAN filament, it decreases between 50.97% and 70.12% (Figure 6). Among the developed porous PAN/NC filaments and in the case of 50% stretching, 0.4 wt.% NC scores the highest fineness strength of 14.21 cN/tex, and 0.8 wt.% NC scores the lowest fineness strength of 9.51 cN/tex. But in the case of 100% stretching, the filament with a ratio of 0 wt.% NC exhibits the highest amount of fineness strength with 11.26 cN/tex, and the filament with a ratio of 0.8 wt.% NC scores the lowest amount of fineness strength with 8.66 cN/tex.

**Figure 7:**
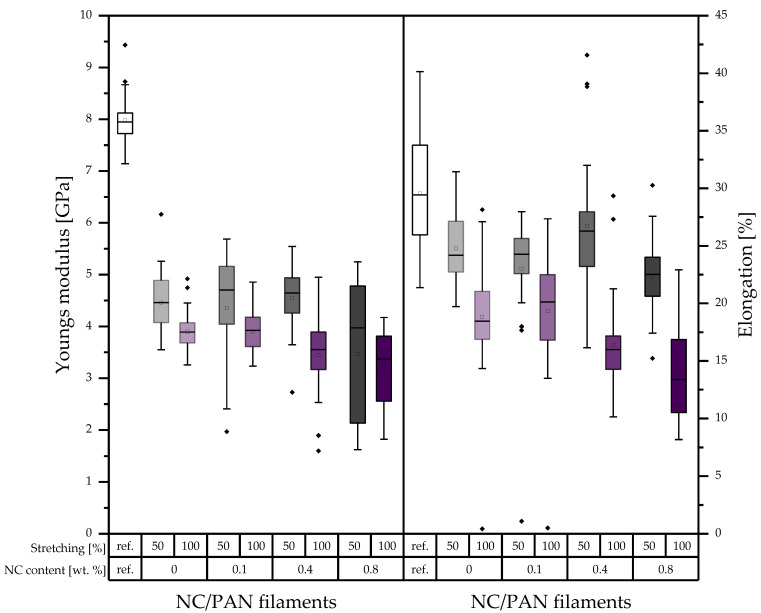
Young’s modulus and elongation of the developed porous PAN/NC filaments (in the boxes: sorted values between 25% and 75%; between the whiskers: values within 1.5 · IQR; central line: median; square: mean; and rhombus: outlier).

In comparison with the reference PAN filament, Young’s modulus of the developed porous PAN/NC filaments decreases between 43.13 and 59.88% as shown in Figure 7. Among the developed porous PAN/NC filaments, 0.4 wt.% NC presents the highest Young’s modulus for 50% stretching and for 100% stretching, and 0 wt.% NC presents the highest Young’s modulus with 4.55 GPa and 3.91 GPa, respectively. Additionally, 0.8 wt.% NC exhibits in both cases the lowest Young’s modulus with 3.45 GPa and 3.21 GPa, respectively.

The reference PAN filaments present the highest elongation in comparison with the developed porous PAN/NC filaments. The elongation decreases between 12.01% and 53.66%. After 50% stretching, 0.4 wt.% NC addition presents the highest elongation of 26.60%. Furthermore, 0.8 wt.% NC scores the lowest elongation of 22.37%. After increasing stretching up to 100%, 0.1 wt.% NC scores the highest elongation of 19.46%. Then, it decreases to the lowest amount at 0.8 wt.% NC with 14.01%. That means that the elongation decreases by increasing the ratios of NC addition and stretching. 

**Figure 8:**
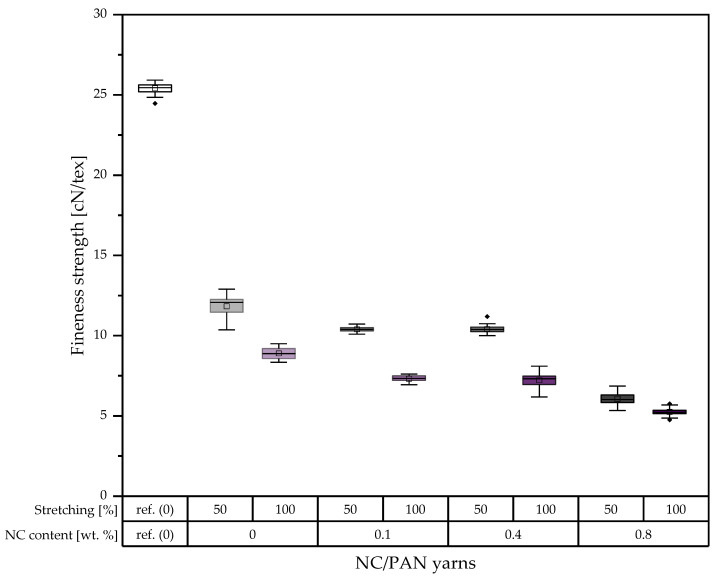
Fineness strength of the developed porous PAN/NC yarns (in the boxes: sorted values between 25% and 75%; between the whiskers: values within 1.5 · IQR; central line: median; square: mean; and rhombus: outlier).

**Figure 9:**
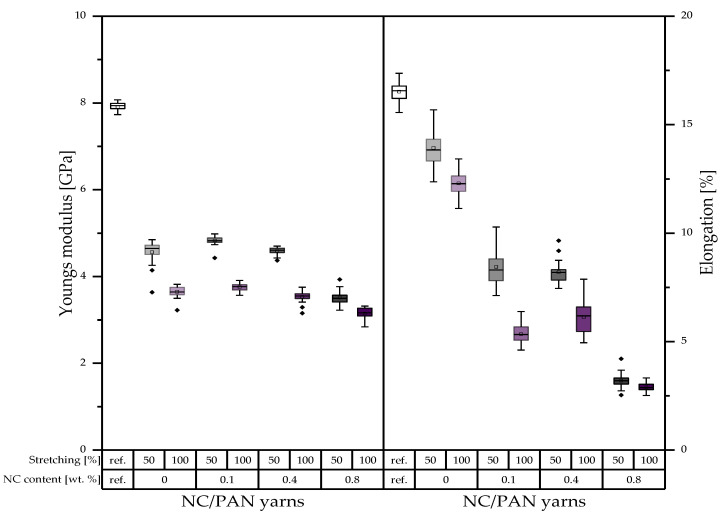
Young’s modulus and elongation of the developed porous PAN/NC yarns (in the boxes: sorted values between 25% and 75%; between the whiskers: values within 1.5 · IQR; central line: median; square: mean; and rhombus: outlier).

The results of mechanical property measurements of the porous PAN/NC yarns (Figure 8 and Figure 9) confirm the previously obtained results in the case of filaments (Figure 6 and Figure 7). The fineness strength of the developed porous PAN/NC yarns decreases between 53.48 and 79.36% in comparison to the reference PAN yarns. Among the developed porous PAN/NC, 0 wt.% NC presents the highest fineness strength in both cases of stretching with 11.83 cN/tex (50% stretching) and 8.90 cN/tex (100% stretching). The porous PAN fibers with NC addition show lower values of fineness strength. 0.1 wt.% and 0.4 wt.% NC exhibits for both stretching ratios similar fineness strengths, while 0.8 wt.% NC has the lowest values with 6.06 cN/tex and 5.25 cN/tex, respectively.

We observed that 0.1 wt.% NC exhibits the highest Young’s moduli with 4.83 GPa for 50% stretching and 3.76 GPa for 100% stretching. The highest elongations of 13.93% and 12.29%, respectively, are in the case of 0 wt.% NC for both stretching ratios. The lowest values for all properties are in the case of 0.8 wt.% NC in both cases of stretching. 

This leads to the conclusion that the mechanical properties have been negatively affected by NC addition and stretching. In all cases, the high stretching ratio of 100% leads to worse mechanical properties. As mentioned before, the higher forces at higher stretching ratios lead to more gaps, whereas the whole fiber structure weakens by the lack of crystalline areas along the filament. Additionally, the formed pores can function as a source for cracks [36]. 

The effect of the addition of NC, on the other hand, is not completely negative. Especially for the 50% stretching ratio, the mechanical properties are stable or slightly increase up to 0.4 wt.% NC and decrease just at 0.8 wt.% NC. For 100% stretching, most of the properties already decrease at 0.4 wt.% NC. The results show that the NC up to a ratio of 0.4 wt.% can also improve the mechanical properties of the porous PAN/NC fibers, as the NC addition equals the negative effect of the increased porosity caused by the NC itself and stretching. These improvement possibilities of the mechanical properties by NC were already reported before [17,18,21]. They also showed an enhancement of the mechanical properties of the final CF and the positive effect of the NC on the stabilization of the PAN [19]. That highlights the role of stabilization and carbonization processes, which can enhance the mechanical properties in a future study to ensure, on the one hand, the necessary textile physical properties and, on the other hand, great functionalization potential by the received high surface area and pore volumes.

### 3.5. Conclusion and Outlook

This study highlighted the importance of the wet spinning technique to produce PAN/NC porous fibers as precursors for carbon fiber manufacturing. Four ratios of NC (0 wt.%, 0.1 wt.%, 0.4 wt.% and 0.8 wt.%) have been added to PAN/DMF (18/82) wt.% spinning solution. During the spinning process in a coagulation bath of DMF/H₂O (85/15) wt.% at 30 °C, two different jet stretching ratios (50% and 100%) were applied to the spun fibers. Measurements based on BET and DFT methodology showed that the porosity of the developed porous PAN/NC fibers was clearly enhanced. The surface area increased by a factor between 9.14 and 13.26 and the total pore volume by a factor between 10.0 and 16.3. The changes in the structure can be seen in SEM pictures of the surface and the cross-section of the fibers. The diameters of the developed porous PAN/NC fibers increased between 13.98 and 42.27% in comparison with the diameter of reference PAN fiber (without NC additives and stretching). The content of NC additives and the stretching processes negatively affected the mechanical properties (fineness strength, Young’s modulus and elongation) of the developed porous PAN/NC filaments, which have generally decreased. In particular, stretching with a ratio of 100% led to worse properties. In the case of 50% stretching, an NC addition up to 0.4 wt.% did not had a negative effect. The fibers with 0.4 wt.% NC presented the best obtained fineness strength (14.21 cN/tex) and Young’s modulus (4.55 GPa). These fibers exhibit the highest pore volume and surface area too. They combine the benefits of the obtained porosity with mechanical properties, which were the least weakened by stretching and NC addition.

As an outlook of this study, stabilization and carbonization processes of the obtained porous PAN/NC fibers should be investigated in a future study in order to enhance the mechanical properties. It is also highly recommended to depend on bio-based additives, the wet spinning method and porous strategy in the production of CF precursors, which could provide better properties for more applications.

## Figures and Tables

**Table 1:** Stretching process and NC additives for and diameter (measured with Axio Imager.M1m Microscope (Jena, Germany) of the fiber cross-section) of the resulting porous PAN/NC fibers.

Sample	Stretching (%)	NC (wt.%)	Diameter of Resulting Fiber (µm)
PAN 0 (control)	0	0	14.74 ± 1.18
pPAN 1_0	50	0	17.78 ± 1.94
pPAN 1_0.1	50	0.1	17.96 ± 1.85
pPAN 1_0.4	50	0.4	17.38 ± 2.75
pPAN 1_0.8	50	0.8	18.97 ± 2.31
pPAN 2_0	100	0	16.80 ± 1.49
pPAN 2_0.1	100	0.1	19.06 ± 1.75
pPAN 2_0.4	100	0.4	18.80 ± 3.39
pPAN 2_0.8	100	0.8	20.97 ± 2.99

## Data Availability

The data presented in this study are available on request from the corresponding author.
